# Evaluating a digital tool for supporting breast cancer patients: a randomized controlled trial protocol (ADAPT)

**DOI:** 10.1186/s13063-019-3971-6

**Published:** 2020-01-15

**Authors:** Emma Lidington, Sophie E McGrath, Jillian Noble, Susannah Stanway, Amanda Lucas, Kabir Mohammed, Winette van der Graaf, Olga Husson

**Affiliations:** 1 The Royal Marsden Hospital, London, United Kingdom; 2 Discover at Imperial College Health Partners, London, United Kingdom; 3 Netherlands Cancer Institute, Amsterdam, Netherlands; 4 Institute of Cancer Research, Sutton, United Kingdom

**Keywords:** Breast cancer, mHealth, Patient activation, Health-related quality of life, Health resource utilization, Patient-reported outcome measures

## Abstract

**Background:**

There are a growing number of mHealth tools for breast cancer patients but a lack of scientific evidence for their effects. Recent studies have shown a mix of positive and negative impacts on users. Here we will assess the impact of OWise Breast Cancer, a mobile application for self-monitoring symptoms and managing care, on the process of self-management.

**Methods:**

This randomized controlled trial with early stage breast cancer patients will assess the effect of OWise use on patient activation at 3 months from diagnosis measured by the PAM-13 questionnaire. We will also assess differences in changes in health-related quality of life, psychological distress, health status, and National Health Service (NHS) health resource utilization over the first year from diagnosis. Participants will be randomly allocated (1:1) to standard care or standard care plus OWise. Participants will complete questionnaires before starting anti-cancer treatment and at 3, 6, and 12 months from diagnosis. Clinical and patient-reported outcome data will be linked to health resource utilization data from Discover, an integrated care record of primary, secondary, and social care in North West London. We will measure contamination in the control group and adjust the sample size to mitigate the dilution of effect estimates. A per-protocol analysis will be conducted as a sensitivity analysis to assess robustness of the primary results.

**Discussion:**

This study aims to generate evidence for the effectiveness of OWise at improving patient activation for women with early-stage breast cancer. The results will show the impact of using the tool at the patient level and the NHS health system level. The outcomes of the study will have implications for the application of OWise across the NHS for breast cancer patients and expansion into other tumor types. Assessing publicly available mHealth tools poses a challenge to trialists due to the risk of contamination. Here we apply various methods to measure, mitigate, and assess the effects of contamination.

**Trial registration:**

The study was registered at clincaltrials.gov (NCT03866655) on 7 March 2019.

## Background

Breast cancer is the most common form of cancer diagnosed in the United Kingdom (UK), with around 55,200 new cases each year [1]. In 2010, the National Health Service (NHS) in England spent around £675 million for the care of patients with breast cancer, with current NHS and broader societal costs likely exceeding this value [2]. With the incidence of breast cancer expected to increase in the UK over the next 15 years [1], the NHS is promoting increased self-management of care [3].

Patients and providers are looking to mHealth applications for potential self-management benefit in cancer populations [4]. mHealth is the application of mobile technology by patients or health care providers to monitor health and improve outcomes [5]. A recent review has found 12 studies assessing mHealth tools to support self-management in breast cancer patients [6]. Many of the studies assessing mHealth tools found promising results with a wound monitoring application reducing health resource utilization [7], an electronic daily journal stabilizing daily functional activity [8] and an application providing information and support improving self-efficacy and quality of life and reducing symptom interference [9]. However, one application providing tailored information before surgery increased levels of anxiety and depression in patients [10]. The inconsistency in effects highlights the need to rigorously assess the impact of mHealth tools before encouraging use.

OWise Breast Cancer is a new mHealth technology for the self-management of care in breast cancer patients. OWise provides tailored medical information, a tracker to self-monitor symptoms and functions to manage care including an appointment calendar, modifiable question list and consultation recording device [11]. OWise, listed in the NHS Apps Library, is freely available for download [12]. OWise was developed outside an academic setting but followed the mHealth development and evaluation process defined by Whittaker et al. [13]. Programmers designed the tool in an iterative process with patients and conducted thorough user testing.

A qualitative study evaluating OWise in the Netherlands showed patients and providers found the tool usable and felt it had the potential to help patients take in more information from consultations, manage appointments, and feel more in control during treatment [14]. To understand the impact of OWise on health behaviors, health-related quality of life (HRQoL), and NHS resource utilization, comparative data are needed.

### Conceptual model

**Fig. 1:**
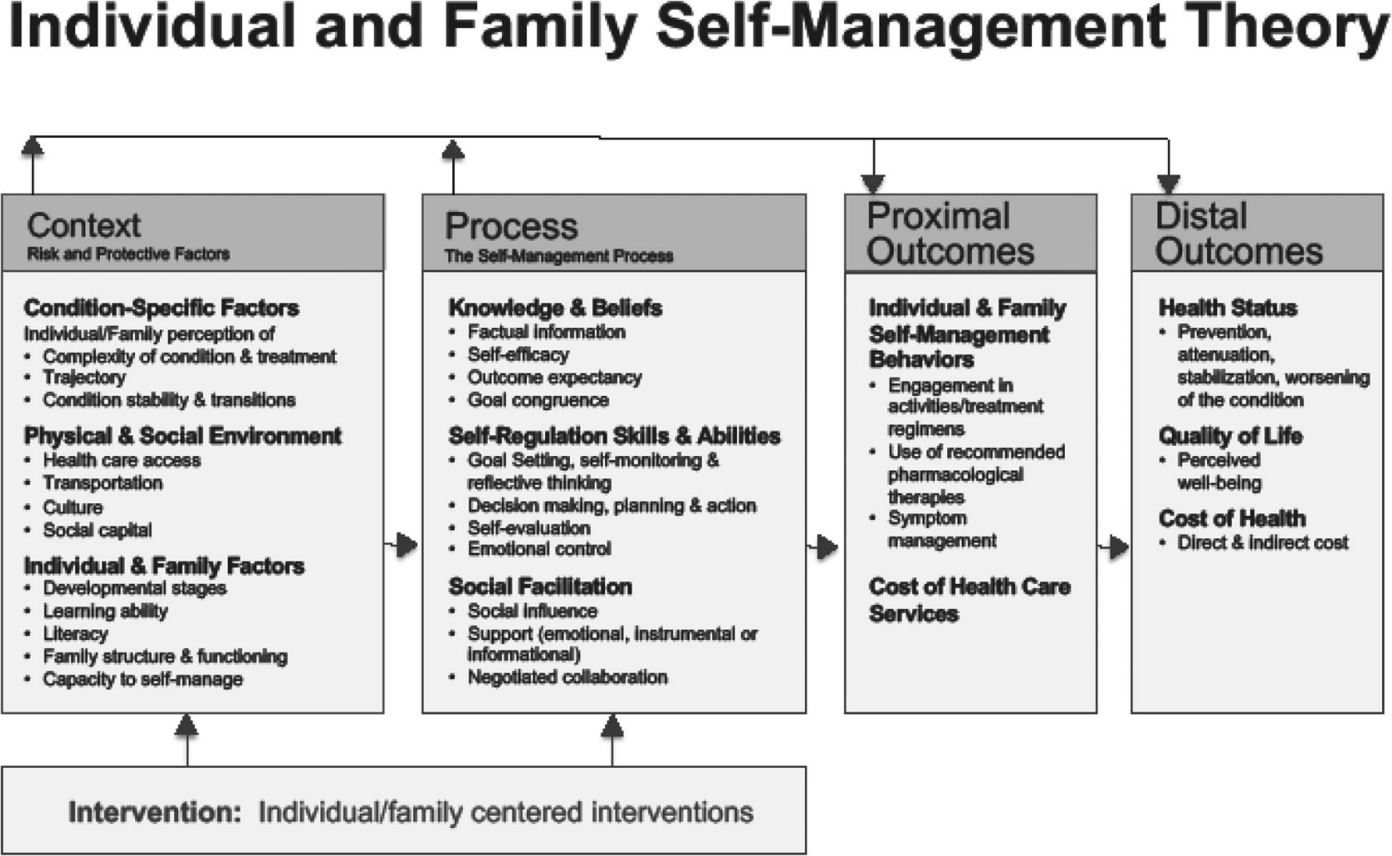
The Individual and Family Self-Management Theory

The conceptual model for OWise is based on the Individual and Family Self-Management Theory. This theory posits that self-management is the process by which individual and family health knowledge and behaviors are used to reach certain health outcomes (Fig. 1) [15]. The theory takes into account individual, medical, social, and environmental factors that influence the process of self-management.

OWise aims to improve HRQoL and reduce health resource utilization by intervening on the self-management process. The digital tool aims to increase knowledge and beliefs by providing tailored medical information and recommended questions in the modifiable question list. OWise aims to improve self-regulation skills and abilities with the symptom tracker and appointment calendar. The proximal outcome we will measure is patient activation and distal outcomes are HRQoL, psychological distress, health status, and health care costs. Studies have previously linked patients’ activation to better HRQoL, improved care experiences, and lower use of NHS resources [16–18].

## Methods

### Aim

This study aims to understand the impact of OWise on health behaviors, HRQoL, and health care utilization in early stage breast cancer patients compared to standard care alone.

### Study design

**Fig. 2:**
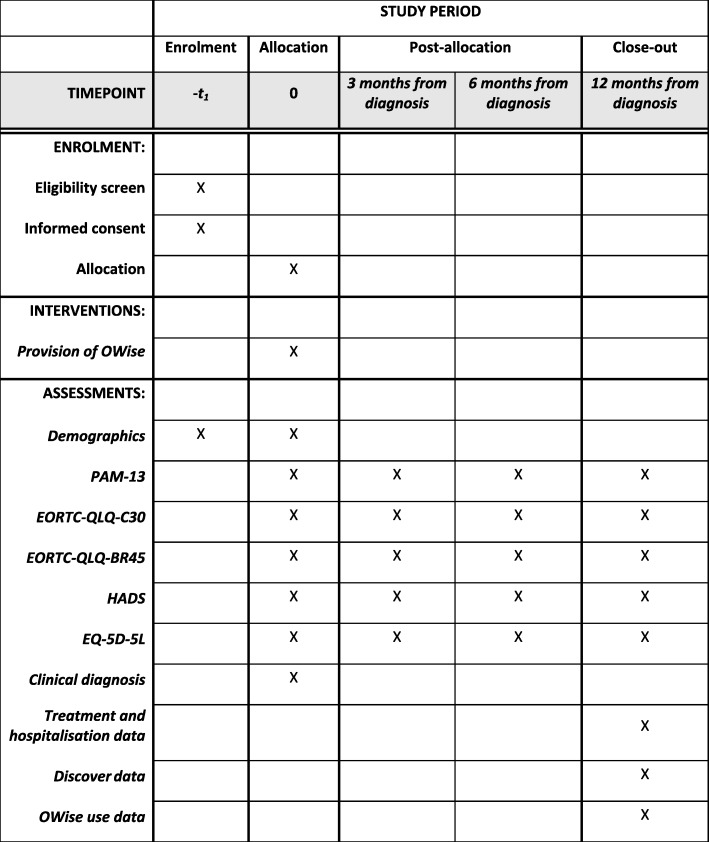
Schedule of enrolment and assessments

We will evaluate the effectiveness of OWise using a multi-center, individually randomized, parallel controlled trial recruiting 122 patients. The intervention group will receive OWise plus standard care, while the control group will receive standard care alone to assess superiority. Due to the nature of the digital tool, it is not possible to blind participants or providers. Outcomes are reported directly by participants and analysis depends on the randomly assigned group. Therefore, outcome assessors and data analysts are not blinded either. Patients in both groups will complete patient-reported outcome measures (PROMs) to assess outcomes at baseline, 3, 6, and 12 months from diagnosis. See Fig. 2 for the SPIRIT diagram showing the schedule of enrolment and assessment and Additional file 1 for the SPIRIT checklist.

### Randomization

Patients will be randomly assigned (1:1) to the intervention or control group [19, 20]. Randomization will be stratified by age group and center. Age is grouped by (1) under 60 years old and (2) 60 years old and over as internet access drops between age groups 45–55 and 55–65 [21] and the incidence of breast cancer in the UK is evenly distributed around age 60 [1]. The Institute of Cancer Research Clinical Trials and Statistics Unit Randomisation Service will generate the randomization sequence and allocate the group by phone.

### Participants

Females (aged 18 years or over) newly diagnosed with early stage breast cancer as a first primary diagnosis will be eligible to take part. Eligibility was restricted to early stage and first primary diagnoses as metastatic patients may have confounding care and psychosocial experiences and patient activation naturally increases with time after a breast cancer diagnosis [22]. Patients must complete the baseline measure before starting anti-cancer treatment. All participating sites are located in the UK, a list of which can be found on the registration website. Exclusion criteria include private care, difficulty reading in English, significant cognitive impairments or poor mental health, and no Internet access.

### Intervention group

OWise is an mHealth tool accessible online or by mobile application [11]. The tool offers tailored medical information, a modifiable question list with tailored recommended questions, a medical terms glossary, useful links to local resources, and a tracking tool for symptoms, an appointment calendar, and a consultation recording device.

In the study, only patients randomized into the intervention group will receive information about OWise. The individual enrolling the patient will provide instructions for creating an account and navigating the tool. Participants will be free to use the tool as much as they wish to mimic real-world use of the application. The tool is free to download and accessible to the participant beyond the study period.

### Control group

Participants in the control group will receive all standard information including leaflets and links to resources that patients usually receive at the time of a new breast cancer diagnosis. Participants in the control group will not be given information about the tool but will also not be explicitly prohibited from using the tool.

### Primary objective

The primary objective of this study is to test whether the use of OWise increases patient activation scores at 3-month follow-up by at least four points more than standard care.

### Secondary objectives

1. To test whether any difference in the change in patient activation between the two groups still exists after controlling for potential covariates.
2. To test whether the use of OWise leads to a smaller decrease in health status at 3-month follow-up than standard care after controlling for potential covariates.
3. To test whether the use of OWise leads to a smaller decrease in HRQoL at 3-month follow-up than standard care alone after controlling for potential covariates.
4. To test whether the use of OWise leads to a lower increase in psychological distress at 3-month follow-up compared to standard care alone after controlling for potential covariates.
5. To test whether the use of OWise reduces the rate of resource utilization in the first year following diagnosis compared to standard care among patients registered in Discover, an integrated health and social care record in North West London.
6. To test whether the use of OWise reduces the average cost per patient in the first year following diagnosis compared to standard care among patients registered in Discover.
7. To describe the change in patient activation in the intervention group compared to the control group in the first year following diagnosis.
8. To describe the level of OWise uptake in the intervention group in the first year following diagnosis.
9. To describe the change in the pattern of patient activation, HRQoL, psychological distress, and health status in the intervention group compared to the control group in the first year following diagnosis.

### Procedure

#### Recruitment

We will continuously sample all patients meeting eligibility criteria diagnosed within the recruitment period. A member of the clinical team will identify eligible patients in multi-disciplinary team meetings or clinic lists and invite potential participants at diagnosis. The name of the digital tool will not be disclosed when inviting patients. If a patient shows interest, a researcher will provide further information either in person or over the phone. Patients can decide to take part any time before starting anti-cancer treatment.

After meeting eligibility criteria and providing written informed consent, participants will be randomized. The researcher will inform the participant of their allocation and provide instructions for accessing the online PROM collection tool. Participants in the intervention arm will be required to complete the baseline measure before using OWise.

### Measures

#### Primary outcome measure

##### Patient Activation Measure (PAM-13)

Patient activation describes the knowledge, skills, and confidence a person has in managing their health and care [23, 24]. The PAM-13 is a 13-item questionnaire that measures patient activation [25]. Each item has four response options from (1) ‘strongly disagree’ to (4) ‘strongly agree,’ and ‘not applicable.’ PAM-13 scores will be calculated according to the guidelines [25]. Scores range on a scale of 1–100 corresponding to four activation levels: 1 (≤ 47.0) not believing activation important, 2 (47.1–55.1) a lack of knowledge and confidence to take action, 3 (55.2–67.0) beginning to take action, and 4 (≥67.1) taking action [25]. This measure has been used widely among cancer patients and across the UK [26–28] and has robust evidence of reliability and validity [23, 29, 30]. Patient activation, as measured by the PAM-13, can be targeted by interventions and change over time [24]. Previous work has shown that higher patient activation is associated with better HRQoL and lower health care utilization [16–18, 31].

#### Secondary outcome measures

##### The European Organisation for Research and Treatment of Cancer Core Quality of Life Questionnaire (EORTC QLQ-C30 version 3) and Updated Breast Cancer Module (EORTC QLQ-BR45)

The EORTC QLQ C-30 is a 30-item instrument measuring HRQoL with five functional scales (physical, role, cognitive, emotional, and social), a global quality of life scale, eight symptom scales or items (fatigue, pain, nausea and vomiting, dyspnea, loss of appetite, sleep disturbance, constipation and diarrhea) and a single item assessing perceived financial impact [32]. The EORTC QLQ-BR45 contains five functional scales or items (body image, future perspective, sexual functioning, sexual enjoyment, and breast satisfaction) and seven symptom scales or items (systemic therapy side effects, upset by hair loss, arm symptoms, breast symptoms, endocrine therapy symptoms, skin mucosis symptoms, and endocrine sexual symptoms) [33]. Scores will be calculated according to EORTC guidelines [34]. All scores range from 0 to 100. Higher scores on functional scales and global quality of life indicate better function and HRQoL, respectively. Higher scores on symptom scales and items indicate higher symptom burden [35]. The measures have strong evidence of validity and reliability in early stage breast cancer patients and have been used in a number of clinical trials allowing for comparisons [33, 36].

##### Hospital Anxiety and Depression Scale (HADS)

This 14-item questionnaire measures psychological distress with seven items assessing anxiety, seven items assessing depression and the summed total score reflecting the level of psychological distress [37]. Three continuous scales will be calculated (anxiety, depression, and overall psychological distress) according to HADS guidelines [37, 38]. Higher scores indicate more psychological distress [37, 38]. The HADS has evidence of reliability and validity in early stage breast cancer patients [36, 39].

##### EuroQol 5-Dimension 5-Level questionnaire (EQ-5D-5L)

This instrument, assessing health status, consists of five items and a visual analogue scale [40]. The items cover five dimensions (mobility, self-care, usual activities, pain/discomfort, and anxiety/depression). Each dimension has five response levels from ‘no problems’ to ‘extreme problems.’ The visual analogue scale records the patient’s self-rated health from 0 to 100, with the highest score indicating ‘The best health you can imagine’ and the lowest score indicating ‘The worst health you can imagine.’ Responses to each item combine to form a five-digit number that describes the patient’s health state. A corresponding index value will be assigned according to a recent valuation study conducted in England [41]. The EQ-5D-5L has a large base of evidence and for validity and reliability in breast cancer patients and can also be used to conduct economic analyses [42].

##### Health care utilization

Health care utilization will be assessed using data routinely collected in Discover. The Discover-linked dataset includes coded information on health and social care resource utilization of individuals registered with a GP practice in the North West London region. Information is collected about the number and type of appointments from primary, secondary, and social care for each patient between diagnosis and 1-year follow-up.

##### Health care costs

The Discover-linked dataset also provides the current costs of health and social care to Clinical Commissioners in North West London based upon Commissioner local pricing. Information on the cost of each type of appointment is calculated routinely and collated together across health care settings to provide a measure of health and social care utilization of each patient.

##### OWise uptake

With patient informed consent, we will evaluate OWise uptake by reviewing timestamps that indicate logging in or modification of a specific function. This information will allow us to evaluate whether participants use the tool, which function patients use, and how long the tool is used for.

#### Contamination

Patient responses to items at each measurement time point will indicate contamination. A set of 19 items will ask participants to identify the use of supportive care services including self-management mobile phone applications or websites. If a participant says yes, we will ask them to name the source in a free-text box to determine whether or not OWise has been used. Prior to recruitment close, the statistician will assess the level of contamination and increase the sample size commensurately [43].

### Data management

The study steering committee determined that a data monitoring committee was unnecessary for this study, as it poses a minimal risk to patient safety and uses only routinely collected or patient-reported data.

#### Patient-reported outcome measure data

Participants will complete PROMS using PROFILES (Patient-Reported Outcomes Following Initial treatment and Long-term Evaluation of Survivorship), an online PROM collection and data management system developed in the Netherlands and implemented at the Royal Marsden [44]. Follow-up time points will be managed by the Royal Marsden as the coordinating center. PROM responses cannot be viewed by the researcher or clinical team until extracted at the end of the study when it will be linked to other study data by the study identification number.

#### Clinical data

With patient informed consent, researchers will collect relevant clinical data from the local electronic patient record and store it digitally at the Royal Marsden. Clinical data will be extracted at the end of the study and linked to other study data by the study identification number.

#### Health care utilization

Study participants will be identified in Discover by NHS number and flagged with the study identification number. The Imperial College Health Partners Discover team will access a de-identified version of the data to analyze health and social care resource utilization on behalf of the Royal Marsden. Health and social care resource utilization data will be extracted at the end of the study and linked to other study data by the study identification number.

#### OWise engagement

Participants will be provided with a unique invitation code linked to their study identification number to input when creating an OWise account. Timestamp data will be identified by the invitation code and extracted at the end of the study. The data will be linked to other study data by the unique invitation code.

### Sample size

Sample size calculations are based on the change in PAM-13 score from baseline to 3 months. A difference of four points is considered clinically relevant [29, 45]. In a similar study, the mean PAM-13 score at baseline for the intervention group was 61.3 (SD 16.61) and 67.9 (SD 16.85) at 3 months [46]. For the control group, the study reported a mean PAM-13 score at baseline of 62.1 (SD 17.30) and 62.8 (SD 14.94) at 3 months. Based on these findings, this study is planned to detect a mean change difference of 5.90 assuming a common standard deviation of 10.0. Using an 80% power, the study will recruit 47 patients per group. This was calculated using a two-sided test with alpha = 0.05. We will increase the sample size taking 23% attrition at 3 months into account (47/(1–0.23)) to 61 patients per group [47] and, as mentioned above, increase the sample size accordingly if contamination is found near the end of recruitment.

### Analysis

The CONSORT-EHEALTH recommendations for reporting randomized trials for developing and evaluating eHealth interventions will guide trial reports [48]. Primary and secondary outcomes will be assessed using intention-to-treat analysis where all participants are analyzed according to the arm to which they were randomized. We will conduct a sensitivity analysis separately as described below. The data will be analyzed after all patients have completed the 1-year follow-up. Data will be reported descriptively at each time point. Mean and standard deviation or median and range will be reported for continuous outcomes. Frequency and percentage will be reported for categorical outcomes.

The association between potential covariates and the primary and secondary endpoints will be explored using univariate analysis. Any variables with a *p* value of < 0.1 will be included in the multivariable model. Multivariable analysis controlling for potential covariates associated with the particular outcome will be conducted using logistic regression for binary outcomes and multiple linear regression for continuous outcomes. Two-sided *p* values of < 0.05 will be considered statistically significant.

#### Primary endpoint

We will compare the PAM-13 score change between the intervention arm and the control arm using independent *t* test. Data will be log-transformed to achieve normality as appropriate. We will also compare the mean change in PAM-13 score in the intervention and control arm in a multiple linear regression model including potential covariates.

#### Secondary endpoints

We will compare the mean change in EQ-5D-5L index score and visual analogue score, EORTC QLQ-C30 and EORTC QLQ-BR45 scale scores in the intervention and control arm in simple and multiple linear regression models including potential covariates.

We will compare the mean change in the three HADS scale scores in the intervention and control arm in simple and multiple linear regression models including potential covariates. Based on the continuous overall psychological distress score, patients are classified as ‘distressed’ when they have a score of ≥ 8, and ‘not distressed’ when they have a score < 8. Frequency, percentages, and any appropriate 95% confidence intervals of this dichotomization at baseline and 3 months will be presented. Chi-square or Fisher’s exact test will be used to compare the level of distress between the intervention and control arms.

We will present the mean rate of resource utilization and cost per patient in the two groups by type of resource (primary, secondary and social care) and for total NHS resources used. Simple and multiple linear regression models including potential covariates will compare the mean rate of total resource utilization and the mean cost per patient in the two groups.

We will describe the average scale scores of the four validated measures in the two groups at the four time points and show graphically the trend in scale scores in each group. We will also compare the mean change in scale scores of the measures across the four time points between the intervention and control arm using a mixed models approach.

To describe OWise uptake, the average number of times logging in at daily intervals throughout the follow-up period will be described and the trend of mean logging in over time will be graphed. We will also show the average frequency of use for each function of the tool over time.

#### Sensitivity analysis

Per-protocol analysis will be performed as a sensitivity analysis to assess the impact of contamination on the primary analysis. In the sensitivity analysis, all participants in the control arm that report using OWise will be excluded. If the sensitivity analysis produces results dissimilar from the primary analysis, we will determine that the primary results are not robust and further research is required.

#### Missing data

Missing data of multi-item scales will be handled according to questionnaire guidelines. Where guidelines are unavailable, items will be mean-imputed if at least half of the items from the scale are answered. Descriptive statistics are based on complete case analysis. We will analyze available data before imputation for the group comparisons and use the complete case data as a form of sensitivity analysis.

### Dissemination

Any protocol modifications will be submitted for approval to the research ethics committee, reflected in the online registration and disseminated by e-mail to site principal investigators and trial coordinators. To mitigate attrition, the coordinating center will engage participants with newsletters via e-mail or post. These will also discuss any changes to study procedures relevant to participants and results of the study. Each party involved will continue to own the data they collected, i.e., the Royal Marsden will own the clinical and PROM data, Discover on behalf of the North West London data custodians will own the health and social care resource utilization data, and Px Healthcare will own the OWise uptake data. The statistician and health economists will have access to the final linked trial dataset. There are no plans to provide public access to the full protocol, participant-level data, or statistical code. The researchers aim to publish results in a peer-reviewed journal and share via social media and conferences. Authorship will be determined by the owner of the data included in the publication.

## Discussion

This study aims to evaluate the impact of OWise on patient activation, psychosocial outcomes, and health and social care utilization. In the face of an expanding mHealth field, robust comparative studies are vital to understand the impact of such tools on patients.

Evaluating mHealth technology available to the public poses a challenge to study design due to the high risk of contamination. To mitigate contamination, we will not disclose the specific tool to participants unless randomized to the intervention group. We felt individual randomization was appropriate over cluster randomization as health care providers are not directly involved in the administration of the intervention and OWise use in the UK is low [43]. Specific items in the questionnaires will measure contamination at each time point. The items will ask patients to identify any supportive care tools or information sources used, including websites and mobile phone applications, with free-text boxes.

Previous work suggests adjusting the sample size for expected contamination [43]. However, no previous literature has reported contamination levels in similar studies. We decided instead to assess the level of contamination before the end of recruitment and increase the sample size if necessary. To assess the impact of contamination on effect estimates, we will also conduct a sensitivity analysis using per-protocol analysis [49]. This will test the robustness of our primary intention-to-treat analysis, the gold standard method for randomized controlled trials [50].

This study will use a new web-based PROM collection tool implemented by the Royal Marsden called PROFILES [44]. Electronic capture of PROMS provides a number of benefits including flexibility for participants, more accurate and timely data collection and reduced time and costs to conduct research [51]. There is also growing evidence for equivalence between paper and electronic PROM collection [52].

This study design may be limited by relying on patient-reports of OWise use to measure contamination. However, this is unavoidable due to data protection arrangements of the tool. Participants may also use similar mHealth tools, which could confound the results. The open-ended items assessing contamination will measure the use of other tools and enable us to control for these effects in analysis as much as possible. This study will also be limited by updates of the application. Post hoc analysis of differences before and after the updates will allow us to assess whether changes in the application reduce or enlarge any effects or change participant uptake.

This study will allow us to assess whether OWise, a patient-focused mHealth technology, can have an impact on self-management processes, HRQoL, and NHS health resource utilization. With the comparative nature of the study and conduct in the NHS system, this will have broad implications for the adoption of this tool by the NHS in future. If successful, this application can be modified to meet the needs of other tumor groups. This study is also applying new methods in a growing field of mHealth evaluation and can serve as an example for researchers in future.

## Trial status

Protocol version 2.0 31/05/2019 was approved on 11/07/2019 with recruitment pending. End of recruitment is planned for 30 June 2020.

## Supplementary information

**Additional file 1:** SPIRIT Checklist.

## Data Availability

Not applicable.

## Abbreviations

EORTC QLQ-BR45: European Organisation for Research and Treatment of Cancer Breast Cancer Quality of Life Questionnaire
EORTC QLQ-C30: European Organisation for Research and Treatment of Cancer Core Quality of Life Questionnaire
EQ-5D-5L: EuroQol 5-Dimension 5-Level questionnaire
HADS: Hospital Anxiety and Depression Scale
HRQoL: Health-related quality of life
NHS: National Health Service
PROM: Patient-reported outcome measure
UK: United Kingdom
